# The predictive and prognostic value of low skeletal muscle mass for dose-limiting toxicity and survival in head and neck cancer patients receiving concomitant cetuximab and radiotherapy

**DOI:** 10.1007/s00405-020-05972-2

**Published:** 2020-04-25

**Authors:** L. F. J. Huiskamp, N. Chargi, L. A. Devriese, P. A. de Jong, R. de Bree

**Affiliations:** 1 Department of Head and Neck Surgical Oncology, UMC Utrecht Cancer Center, University Medical Center Utrecht and Utrecht University, Heidelberglaan 100, 3584 CX Utrecht, The Netherlands; 2 Department of Medical Oncology, University Medical Center Utrecht and Utrecht University, Heidelberglaan 100, 3584 CX Utrecht, The Netherlands; 3 Department of Radiology and Nuclear Medicine, University Medical Center Utrecht and Utrecht University, Heidelberglaan 100, 3584 CX Utrecht, The Netherlands; 4 Department of Head and Neck Surgical Oncology, University Medical Center Utrecht, House Postal Number Q.05.4.300, PO BOX 85500, 3508 GA Utrecht, The Netherlands

**Keywords:** Sarcopenia, Dose-limiting toxicity, Cetuximab, Skeletal muscle mass, Head and neck cancer, Computer-assisted image analysis

## Abstract

**Purpose:**

This study aims to investigate the predictive value of low skeletal muscle mass (SMM) for cetuximab dose-limiting toxicity (DLT) and its prognostic value in head and neck squamous cell carcinoma (HNSCC) patients treated with concomitant cetuximab and radiotherapy.

**Methods:**

Patients diagnosed with HNSCC and treated with primary or adjuvant concomitant cetuximab and radiotherapy were included. Clinical and demographic variables were retrospectively retrieved and SMM was measured at the level of the third cervical vertebra using pre-treatment diagnostic computed tomography or magnetic resonance imaging. An optimal cut-off value for low SMM was determined based on the lowest log-likelihood associated with cetuximab DLT. A multivariate linear regression model was used to determine predictive factors for cetuximab DLT. The prognostic value of low SMM for disease-free and overall survival was analyzed using Kaplan–Meier curves.

**Results:**

The optimal cut-off value for low SMM as a predictor of cetuximab DLT was an LSMI ≤ 45.2 cm^2^/m^2^. Of the 91 included patients, 74.7% had low SMM and 30.8% experienced cetuximab DLT. At multivariate analysis, low SMM had no predictive value for DLT (OR 0.83; 95% CI 0.27–2.56; *p* = 0.74). The Kaplan–Meier curve demonstrated that patients with low SMM had significantly lower overall survival (Log Rank *χ*^2^ = 5.87; *p* = 0.02).

**Conclusion:**

Low SMM is highly prevalent in HNSCC patients treated with concomitant cetuximab and radiotherapy. Low SMM has no predictive value for cetuximab DLT in HNSCC patients. Low SMM is probably not a prognostic factor for overall survival in highly selected HNSCC patients treated with concomitant cetuximab and radiotherapy and unfit for platin-based chemotherapy.

## Introduction

Head and neck cancer is the sixth most common cancer, with over 600,000 new cases annually worldwide [1]. At diagnosis, locoregionally advanced disease is present in up to 60% of patients [1]. Locoregionally advanced-stage head and neck squamous cell carcinoma (HNSCC) is generally treated with surgery plus adjuvant radiotherapy with or without cisplatin chemotherapy or, as primary treatment, concomitant cisplatin chemotherapy and radiotherapy with salvage surgery in reserve for residual disease or recurrence [1]. The addition of chemotherapy to radiotherapy improves disease control and survival but also results in increased toxicity and can, therefore, influence adherence to the treatment [2]. Cisplatin dose-limiting toxicity (DLT) includes, among others, bone marrow depression, ototoxicity, and nephrotoxicity [3]. This can cause treatment delay, dose reduction, and possible failure to complete treatment as well as decreased quality of life [3].

To improve treatment adherence and reduce toxicity, predictive factors should be identified that indicate the risk of a patient to experience DLT. Currently, patients are evaluated by their oncologist to determine whether they are medically fit to undergo cisplatin treatment. This takes into consideration age, comorbidities, and the presence of contraindications for cisplatin, such as impaired renal function, poor general health, bone marrow suppression, and impaired hearing. If patients are considered unfit for cisplatin alternative options to increase the anti-tumor effect of radiotherapy include the addition of cetuximab [4]. However, patients treated with cetuximab in combination with radiotherapy may also experience considerable amounts of toxicity, specifically leucopenia, neutropenia, and mucositis [5]. Therefore, to improve treatment adherence and reduce toxicity, predictive factors should be identified that indicate the risk of DLT.

Low skeletal muscle mass (SMM) is a possible predictive factor to estimate whether a patient will experience chemotherapy DLT. Moreover, low SMM may also be a prognostic factor. Low SMM has a high prevalence in adults with cancer; in HNSCC prevalence as high as 55% has been reported [3]. SMM can be measured on a routinely performed computed tomography (CT) or magnetic resonance imaging (MRI) of the head and neck [6–8].

Low SMM has previously been linked to an increased prevalence of chemotherapy DLT for several types of cancer such as breast [9], colorectal [10], renal [11], lung [12], and oesophago-gastric cancer [13]. Specifically for HNSCC, Wendrich et al*.* demonstrated that low SMM is a predictive factor for platin DLT (occurring in 30.4%) in patients treated with platin-based chemotherapy and radiotherapy [3].

Based on previous evidence supporting the predictive value of low SMM for chemotherapy DLT in several types of cancer, it is logical to question whether low SMM is also predictive for DLT in treatment of HNSCC using cetuximab. This study focusses on investigating the possible predictive value of low SMM for DLT during concomitant cetuximab and radiotherapy treatment of locally advanced HNSCC. In addition, the prognostic value of low SMM for overall survival (OS) and the disease-free survival (DFS) in HNSCC patients treated with concurrent cetuximab and radiotherapy is investigated.

## Methods

### Ethical approval

The design of this study was approved by the Medical Ethical Research Committee of the University Medical Center Utrecht (approval ID 17-365/C). All procedures in this study were in accordance with the ethical standards of the institutional and/or national research committee and with the 1964 Helsinki declaration and its later amendments or comparable ethical standards.

### Patients and study design

We conducted a retrospective study of HNSCC patients treated with primary or adjuvant concomitant cetuximab and radiotherapy in the University Medical Center Utrecht between January 2007 and December 2018. The included patients were unfit for cisplatin treatment. HNSCC patients were included if they had a pre-treatment (≤ 3 months prior) diagnostic imaging scan (CT or MRI) of the third cervical vertebra (C3) level which was suitable for muscle segmentation. Patients were excluded if treatment was provided with palliative intent. Relevant demographic and clinical variable such as age at diagnosis, sex, weight, length, body mass index (BMI), alcohol consumption, alcohol abuse as identified by the treating physician, comorbidity as expressed by the Adult Comorbidity Evaluation-27 (ACE-27), tumor, lymph nodes, and metastasis (TNM) staging, treatment regimen, cetuximab DLT data, date of last follow-up, and eventually, the date of recurrent disease or death were obtained from patients records.

### Image analysis and measurements

The cross-sectional area (CSA) of skeletal muscles was measured on pre-treatment diagnostic CT or MRI imaging that included the C3 vertebra. Segmentation of the muscle was performed using the commercially available SliceOmatic (Tomovision, Canada) by a single researcher (L.H.) on the axial slide which showed the entire vertebral arc as well as both transverse processes. The CT scans used were 3-mm axial slices with or without contrast made using Philips (16-slice or 64-slice) or Siemens scanners (40-slice) and the MRI scans were axial T1 weighted sequence without fat suppression made using Philips scanners (1.5 or 3 T). CSA was calculated as the sum of the measured area of both sternocleidomastoid muscles (SCM) and the paravertebral muscles. If tumor growth interfered with the measurement of either the left or right SCM, the area of the contralateral SCM was used to replace it. Patients were excluded, if, the CSA could not be measured reliably due to a CT or MRI artifacts, a too small field of view, or tumor growth in both SCM.

**Fig. 1:**
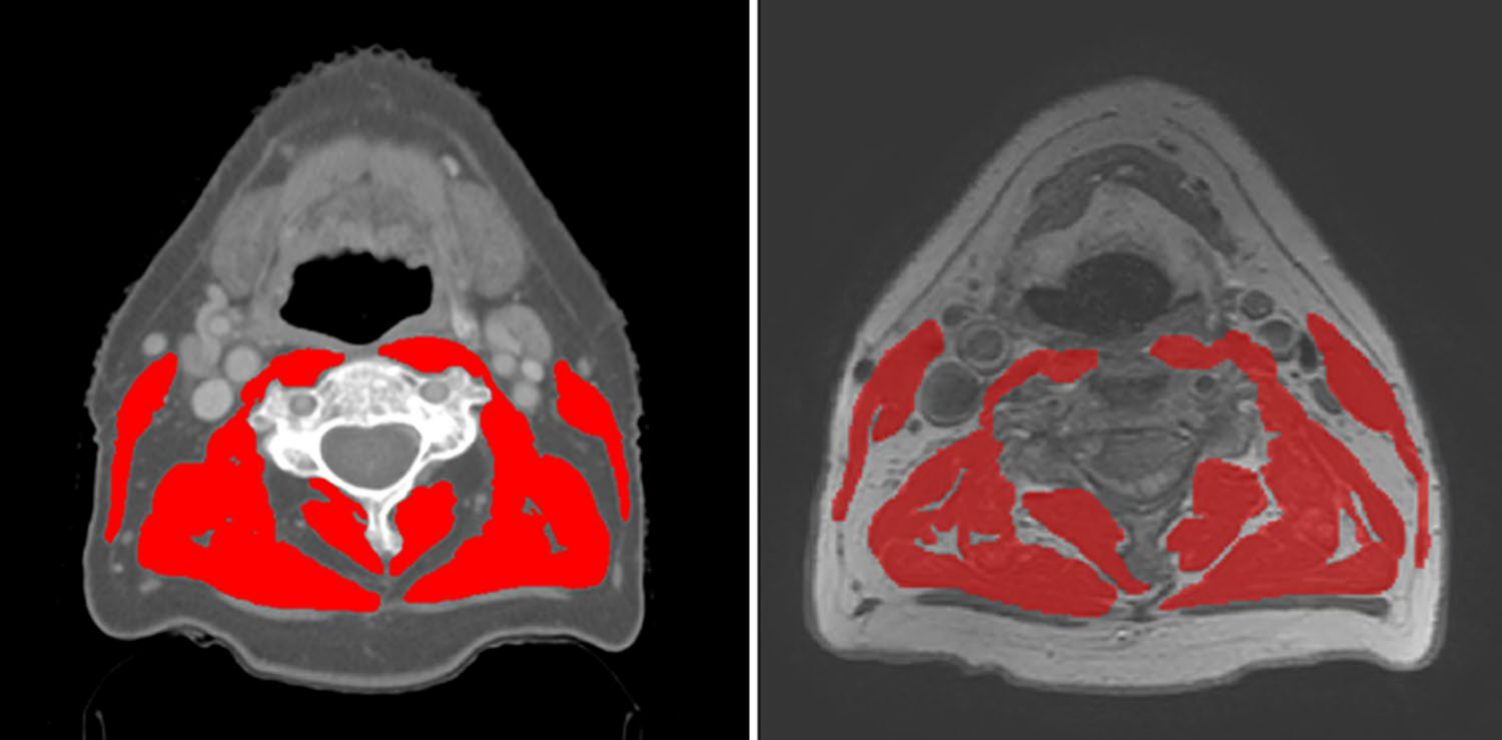
Example of delineation on 3 mm axial slide of CT (Siemens 40-slice) (left) and axial T1 weighted sequence MRI (Philips 1.5 T) (right) at the level of C3 using SliceOmatic. The left and right SCM as well as the paravertebral muscles are delineated excluding the trapezius muscle. Please note that the muscles in the anterior neck are not included in the delineation as previously described

In the case of CT imaging, muscle area was measured semi-automatic using a combination of manual segmentation in a predefined radiodensity range of − 29 to + 150 Hounsfield units (HU) [14]. In the case of MRI imaging, muscle area was measured manually. Figure 1 shows an example of muscle delineation at the C3 level. The CSA at C3 level was converted to the CSA at third lumbar vertebra L3 level using the formula previously published by Swartz et al*. *[7]. The CSA at L3 level was corrected for squared height to create the lumbar skeletal muscle index (LSMI).

### Dose-limiting toxicity

DLT was defined as any toxicity that resulted in a treatment postponement of ≥ 4 days, dose reduction of ≥ 50%, dose omission, or termination of cetuximab treatment before completing the predetermined cetuximab regimen (most commonly consisting of eight cycles, none extending beyond last radiotherapy fraction).

### Survival

OS was defined as the time between the date of diagnosis and the date of death or the date of the last follow up. DFS was defined as the time between the date of diagnosis and the date of recurrence or the date of the last follow up.

### Statistical analysis

Statistical analysis of the data was performed using IBM SPSS statistics 25. Descriptive statistics for categorical variables were presented as frequencies and percentages. Continuous variables with normal distribution were presented as mean with standard deviation (SD), while those with skewed distribution were presented as median with interquartile range (IQR).

The means of the continuous variables with the presence or absence of low SMM were computed using the independent sample t-tests. The percentages of the categorical variables with the presence or absence of low SMM were analyzed using the Pearson’s or Mantel–Haenszel chi-square test. The risk parameters were calculated and presented with corresponding 95% confidence intervals (95% CI) and *p* values.

The predictive value of low SMM on cetuximab DLT was evaluated using univariate and multivariate logistic regression. A Cox proportional hazard regression model was used for univariate and multivariate analysis of OS and DFS. Covariates used in the multivariate analysis were selected based on the clinical significance or statistical significance (*p* < 0.05) in univariate cox or logistic regression analysis. Statistical significance was evaluated at the 0.05 level using 2-sides tests. OS and DFS were visualized using Kaplan–Meier survival curves and number at risk tables.

## Results

### Study population

**Fig. 2:**
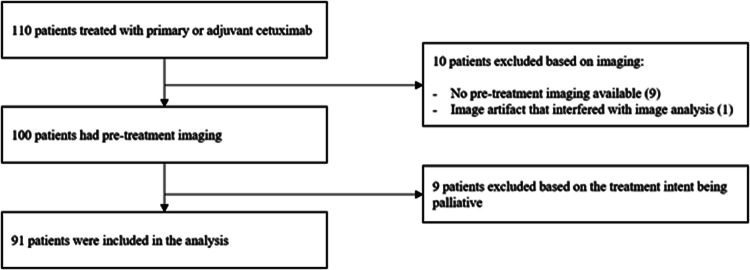
Flowchart of patient inclusion

Between 2007 and 2018, 110 HNSCC patients were treated with primary or adjuvant cetuximab and radiation for oropharynx, hypopharynx, or larynx tumor. Of these patients, 100 had pre-treatment imaging of the C3 vertebra which is necessary for the determination of SMM. Additionally, patients receiving cetuximab with palliative intent were excluded. As can be seen in Fig. 2, 91 patients were included in the analysis, 28 patients (30.8%) experienced cetuximab DLT and 63 (69.2%) experienced no cetuximab DLT.

### Determining the optimal cut-off value for low SMM

The cut-off value for low SMM was determined by calculating the log-likelihood using a technique previously described by Williams et al. [15]*.* The cut-off value best associated with the presence of cetuximab DLT (lowest Log-Likelihood value) was LSMI ≤ 45.2 cm^2^/m^2^. Using this cut-off value for the study population, 68 (74.7%) were identified with low SMM and 23 (25.3%) were identified without low SMM.

### Characteristics of study population

Table 1 shows the general characteristics of the study population according to the presence or absence of low SMM. Significant differences were observed for the occurrence of low SMM in the presence of weight loss six months prior to diagnosis, sex, and body mass index. Patients with low SMM were more likely to be female (35.3% versus 4.3%; *χ*^2^ = 8.26; *p* = 0.002), more likely to have experienced weight loss in the six months prior to diagnosis (50.0% versus 17.4%; MH *χ*^2^ = 9.32; *p* = 0.01), and were less likely to be overweight (BMI 25–29.9) (16.2% versus 39.1%; MH *χ*^2^ = 45.88; *p* < 0.001) or obese (BMI ≥ 30) (2.9% versus 52.2%; MH *χ*^2^ = 45.88; *p* < 0.001).

**Table 1:** General characteristics of the study population according to presence or absence of low SMM

*n*	Low SMM	Without low SMM	*p* value^a^
	68 (74.7%)	23 (25.3%)	
	*n *(%) or mean (± SD)	*n *(%) or mean (± SD)	
Sex			
Female	24 (35.3%)	1 (4.3%)	0.002**
Male	44 (64.7%)	22 (95.7%)	
Age at diagnosis	62.18 (± 7.22)	63.33 (± 7.78)	0.521
Weight loss 6 months prior			
None	34 (50.0%)	19 (82.6%)	0.008*
≤ 10%	17 (25.0%)	4 (17.4%)	
> 10%	17 (25.0%)	0 (0.0%)	
Body Mass Index (kg/m^2^)			
< 20	29 (42.6%)	0 (0%)	< 0.001**
20–24.9	26 (38.2%)	2 (8.7%)	
25–29.9	11 (16.2%)	9 (39.1%)	
≥ 30	2 (2.9%)	12 (52.2%)	
Smoking status			
Non-smoker	2 (2.9%)	3 (13.0%)	0.189
Former-Smoker	23 (33.8%)	8 (34.8%)	
Smoker	43 (63.2%)	12 (52.2%)	
Pack-Years			
0	2 (3.4%)	3 (14.3%)	0.621
1–15	8 (13.6%)	2 (9.5%)	
16–25	9 (15.3%)	5 (23.8%)	
26–40	17 (28.8%)	6 (28.6%)	
≥ 41	23 (39.0%)	5 (23.8%)	
Alcohol use			
No	5 (8.5%)	5 (15.6%)	0.205
Former	11 (18.6%)	2 6.3%)	
Yes	43 (72.9%)	25 (78.1%)	
Alcohol (U/day)	4.25 (± 4.19)	2.38 (± 1.69)	0.051
Alcohol abuse			
No	40 (58.8%)	17 (73.9%)	0.443
Yes, current	6 (8.8%)	1 (4.3%)	
Yes, former	22 (81.5%)	5 (21.7%)	
ACE-27 score^b^			
None	6 (8.8%)	2 (8.7%)	0.998
Mild	17 (25.0%)	6 (26.1%)	
Moderate	25 (36.8%)	8 (34.8%)	
Severe	20 (29.4%)	7 (30.4%)	
Tumor site			
Oropharynx	48 (70.6%)	17 (73.9%)	0.731
Hypopharynx	8 (11.8%)	1 (4.3%)	
Larynx	2 (2.9%)	2 (8.7%)	
Other	10 (14.7%)	3 (13.1%)	
TNM-stage			
Stage 1	1 (1.5%)	0 (0.0%)	0.621
Stage 2	2 (2.9%)	1 (4.3%)	
Stage 3	8 (11.8%)	5 (21.7%)	
Stage 4	57 (83.8%)	17 (73.9%)	
Surgery			
No	64 (94.1%)	22 (95.7%)	0.627
Yes	4 (5.9%)	1 (4.3%)	
Recurrence			
No	46 (67.6%)	20 (87.0%)	0.059
Yes	22 (32.4%)	3 (13.0%)	
Synchronous tumor			
No	56 (82.4%)	20 (87.0%)	0.752
Yes	12 (17.6%)	3 (13.0%)	
HPV status^c^			
Negative	44 (64.7%)	12 (52.2%)	0.208
Positive	7 (10.3%)	6 (26.1%)	
Missing	17 (25.0%)	5 (21.7%)	

*Correlation is significant at the 0.05 level (2-tailed)

**Correlation is significant at the 0.01 level (2-tailed)

^a^Chi-square test or Independent sample *t*-test

^b^ACE-27 = Adult Comorbidity Evaluation

^c^HPV = Human Papillomavirus

### Univariate and multivariate analysis

Table 2 shows the univariate and multivariate logistic regression analysis for the association with cetuximab DLT. In the univariate analysis, weight loss six months prior to diagnosis and ACE-27 score had statistically significant predictive value for cetuximab DLT. Low SMM did not show significant predictive value for cetuximab DLT (OR = 0.60; 95% CI 0.22–1.63; *p* = 0.31). The multivariate Cox regression analysis for the association with cetuximab DLT included weight loss, ACE-27 score, and low SMM. These variables were chosen because of their clinical significance or statistical significance in the univariate analysis. Both weight loss six months prior to diagnosis and ACE-27 score showed statistically significant predictive value for cetuximab DLT in this multivariate analysis. Low SMM remained non-significant in multivariate analysis (OR 0.83; 95% CI 0.27–2.56; *p* = 0.74).

**Table 2:** Univariate and multivariate analysis of predictive factors for cetuximab dose-limiting toxicity

Variable	Cetuximab dose-limiting toxicity					
	Univariate analysis^a^			Multivariate analysis^b^		
	OR	95% CI	*p *value	OR	95% CI	*p* value
Sex	0.442	0.169–1.157	0.096			
Age when diagnosed	1.019	0.958–1.084	0.553			
Weight loss 6 months prior						
None	Ref			Ref		
≤ 10%	0.235	0.062–0.896	0.034*	0.199	0.045–0.871	0.032*
> 10%	0.302	0.077–1.178	0.085	0.309	0.068–1.405	0.128
Body Mass Index (kg/m^2^)						
20–24.9	Ref					
< 20	1.350	0.44–4.316	0.613			
25–29.9	2.455	0.719–8.380	0.152			
≥ 30	0.818	0.176–3.804	0.798			
Smoking status						
Non-smoker	Ref					
Smoker	0.563	0.085–3.705	0.550			
Former-Smoker	0.825	0.119–5.710	0.845			
Alcohol use						
No	Ref					
Yes	1.043	0.245–4.431	0.955			
Former	1.037	0.173–6.233	0.968			
Alcohol (U/day)	0.925	0.801–1.070	0.294			
ACE-27 score^c^						
None	Ref			Ref		
Mild	0.188	0.018–0.749	0.023*	0.091	0.012–0.672	0.019*
Moderate	0.125	0.021–0.737	0.022*	0.094	0.014–0.642	0.016*
Severe	0.117	0.019–0.718	0.020*	0.104	0.015–0.740	0.024*
HPV status^d^	0.900	0.216–3.742	0.885			
Low SMM (LSMI ≤ 45.2)	0.603	0.224–1.625	0.317	0.827	0.268–2.556	0.742

*Correlation is significant at the 0.05 level (2-tailed)

**Correlation is significant at the 0.01 level (2-tailed)

^a^Logistic regression analysis

^b^Multivariate logistic regression (Backward Wald model)

^c^ACE-27 = Adult Comorbidity Evaluation

^d^HPV = Human Papillomavirus

Table 3 shows the univariate and multivariate Cox regression analysis for the association with OS. The univariate analysis showed that weight loss six months prior to diagnosis, HPV status, alcohol units per day, and low SMM are statistically significant prognostic factors for OS. These statistically significant prognostic factors of the univariate analysis were used in the multivariate analysis. BMI was close to statistically significant (HR 0.43; 95% CI 0.18–1.01; *p* = 0.05), therefore, BMI was added into the multivariate analysis. With weight loss, BMI, HPV status, alcohol units per day, and low SMM entered into the multivariate analysis, the two statistically significant prognostic factors were weight loss of more than 10% prior to diagnosis (HR 3.66; 95% CI 1.66–8.09; *p* = 0.001) and positive HPV status (HR 0.24; 95% CI 0.07–0.85; *p* = 0.03). Low SMM showed no statistically significant prognostic value in the multivariate analysis (HR 1.48; 95% CI 0.48–4.58; *p* = 0.50).

**Table 3:** Univariate and multivariate analysis of prognostic factors for overall survival

Variable	Overall survival					
	Univariate analysis^a^			Multivariate analysis^b^		
	HR	95% CI	*p* value	HR	95% CI	*p* value
Sex	0.098	0.883–3.203	0.114			
Age when diagnosed	0.973	0.939–1.009	0.141			
Weight loss 6 months prior						
None	Ref			Ref		
≤ 10%	1.681	0.872–3.241	0.121	1.595	0.777–3.273	0.203
> 10%	3.407	1.786–6.500	< 0.001**	3.662	1.658–8.085	0.001**
Body Mass Index (kg/m^2^)						
20–24.9	Ref			Ref		
< 20	1.564	0.849–2.883	0.152	0.964	0.488–1.984	0.964
25–29.9	0.425	0.178–1.013	0.054	0.704	0.344–2.056	0.704
≥ 30	0.593	0.236–1.489	0.266	0.702	0.312–5.628	0.702
Smoking status						
Non-smoker	Ref					
Smoker	6.594	0.898–48.405	0.064			
Former-Smoker	2.776	0.361–21.324	0.326			
Alcohol (U/day)	1.083	1.017	0.013*	1.036	0.969–1.108	0.303
ACE-27 score^c^						
None	Ref					
Mild	1.783	0.497–6.391	0.375			
Moderate	2.743	0.818–9.192	0.102			
Severe	2.324	0.660–8.182	0.189			
TNM-stage						
Stage 1	Ref					
Stage 2	0.600	0.054–6.727	0.679			
Stage 3	0.420	0.049–3.619	0.430			
Stage 4	0.988	0.135–7.200	0.990			
HPV status^d^	0.139	0.033–0.580	0.007**	0.238	0.066–0.850	0.027*
Low SMM (LSMI ≤ 45.2)	2.453	1.159–5.191	0.019*	1.479	0.478–4.575	0.497

*Correlation is significant at the 0.05 level (2-tailed)

**Correlation is significant at the 0.01 level (2-tailed)

^a^Cox regression analysis

^b^Multivariate cox regression (Backward Wald model)

^c^ACE-27 = Adult Comorbidity Evaluation

^d^HPV = Human Papillomavirus

Table 4 shows the univariate and multivariate Cox regression analysis for the association with DFS. The univariate analysis showed that none of the clinically relevant variables had significant prognostic value for DFS. However, BMI (HR 0.22; 95% CI 0.05–1.00; *p* = 0.05) and weight loss six months prior to diagnosis (HR 2.56; 95% CI 0.99–6.57; *p* = 0.05) did demonstrate a *p* value close to statistically significant. Low SMM, BMI, and weight loss six months prior to diagnosis were entered into the multivariate analysis. In the multivariate analysis, none of the entered variables demonstrated a statistically significant prognostic value for DFS.

**Table 4:** Univariate and multivariate analysis of prognostic factors for disease-free survival

Variable	Disease-free survival					
	Univariate analysis^a^			Multivariate analysis^b^		
	HR	95% CI	*p* value	HR	95% CI	*p* value
Sex	1.387	0.553–3.482	0.486			
Age when diagnosed	0.977	0.926–1.030	0.387			
Weight loss 6 months prior						
None	Ref			Ref		
≤ 10%	1.537	0.576–4.102	0.391	1.494	0.547–4.082	0.434
> 10%	2.556	0.994–6.574	0.051	2.036	0.717–5.777	0.182
Body Mass Index (kg/m^2^)						
20–24.9	Ref					
< 20	0.903	0.361–2.260	0.828	0.740	0.283–1.936	0.540
25–29.9	0.221	0.049–1.001	0.050	0.310	0.067–1.439	0.135
≥ 30	0.748	0.237–2.360	0.620	2.211	0.469–10.567	0.320
ACE-27 Score^c^						
None	Ref					
Mild	0.946	0.190–4.714	0.946			
Moderate	1.123	0.241–5.218	0.883			
Severe	1.150	0.242–5.463	0.860			
TNM-stage						
Stage 1	Ref					
Stage 2	0.445	0.028–7.159	0.568			
Stage 3	0.182	0.016–2.011	0.164			
Stage 4	0.448	0.060–3.346	0.434			
HPV status^d^	0.296	0.068–1.280	0.103			
Low SMM (LSMI ≤ 45.2)	2.421	0.722–8.112	0.152	3.786	0.712–20.123	0.118

*Correlation is significant at the 0.05 level (2-tailed)

**Correlation is significant at the 0.01 level (2-tailed)

^a^Cox regression analysis

^b^Multivariate cox regression (Backward Wald model)

^c^ACE-27 = Adult Comorbidity Evaluation

^d^HPV = Human Papillomavirus

### Overall survival and disease-free survival

**Fig. 3:**
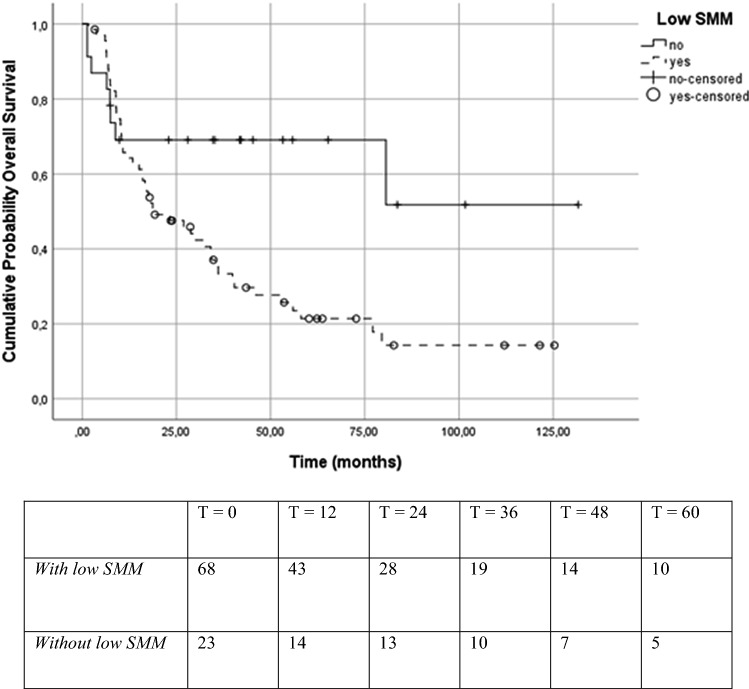
Kaplan Meier curve and number at risk table for patients with and without low SMM for overall survival (log rank *χ*^2^ = 5.8730; *p* = 0.015)

**Fig. 4:**
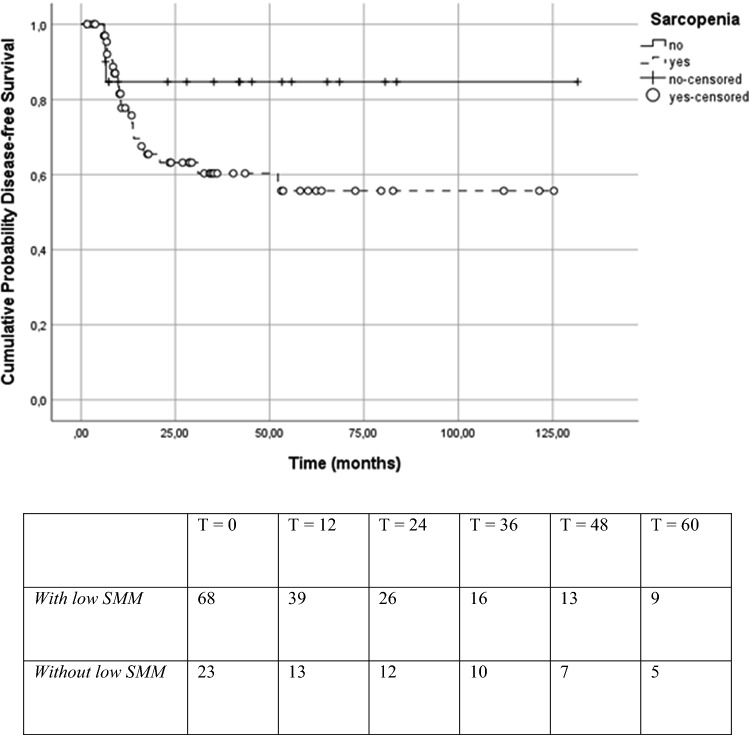
Kaplan Meier curve and number at risk table for patients with and without low SMM for disease-free survival (log rank *χ*^2^ = 2.191; *p* = 0.139)

Figures 3 and 4 show the Kaplan Meier Survival curves and number at risk tables for patients with and without low SMM. As can be seen in Fig. 3, patients with low SMM have a lower median OS (18.48 months; IQR 9.04–40.26) compared to patients without low SMM (34.66 months; IQR 7.39–55.85) (log rank *χ*^2^ = 5.87; *p* = 0.02). As shown in Fig. 4, patients with low SMM did not show a significantly different mean DFS rate (14.83 months; IQR 8.80–35.17) compared to patients without low SMM (28.02 months; IQR 6.51–55.85) (log rank *χ*^2^ = 2.19; *p* = 0.14).

## Discussion

This study demonstrated that low SMM has a high prevalence in HNSCC patients with 74.7% of the patients included in this study. Additionally, roughly a third of the patients (30.8%) experienced cetuximab DLT. This study showed that weight loss of more than 10% in the 6 months prior to diagnosis as well as comorbidities as measured by the ACE-27 have predictive value for cetuximab DLT. However, no significant predictive value of low SMM was observed for cetuximab DLT in HNSCC patients treated with cetuximab and radiotherapy. Furthermore, this study shows that low SMM may be of prognostic value in these patients for overall survival.

The most commonly used method for the measurement of SMM in cancer patients is based on measurement of the CSA of skeletal muscles on a single transversal slice at the level of the third lumbar vertebra (L3) [7]. Swartz et al. found a correlation between the CSA of skeletal muscles at C3 and L3 (*r* = 0.785) [7]. Using a multivariate prediction equation, the correlation between measured the CSA at L3 and estimated CSA at L3 from C3 was even stronger (*r* = 0.895). Therefore, the CSA of skeletal muscles at the level of C3 can be used as an alternative to that of L3 to assess total SMM in patients who only received imaging of the head and neck area [10]. Moreover, an excellent inter-observer agreement for measurement of skeletal muscle CSA was found [8]. Additionally, a recent study demonstrated a strong correlation (*r*^2^ = 0.94, *p* < 0.01) between the measurement of CSA on CT imaging and MRI imaging [6]. Measurement of skeletal muscle CSA at the level of L3 can, therefore, be assessed using skeletal muscle CSA measurement at the level of C3 on CT or MRI.

Several studies show that low SMM is a prognostic factor in HNSCC patients [16–18]. In the present study, low SMM showed no significant prognostic value in the multivariate cox regression analysis.. The populations in studies showing low SMM as a prognostic factor consisted of elderly [16], cisplatin fit [17] and advanced-stage disease patients [18]. Some reasons for this difference in a prognostic value of the present study with highly selected patients compared to other studies can be hypothesized. First, this study consists of a limited number of patients. Secondly, these patients were unfit for cisplatin-based chemotherapy, mainly because of comorbidity which can affect overall survival as well. The poor condition of these selected patients is illustrated by the very high prevalence of low SMM. Finally, in our study only patients with locoregional advanced stage disease with generally already a poor prognosis were included.

This is the first study on the predictive value of low SMM for cetuximab DLT in HNSCC patients. There is only one study that previously looked at the predictive value of low SMM for DLT in cancer patients treated with cetuximab [10]. In this study, Barret et al. showed that in metastatic colorectal cancer patients treated with cetuximab low SMM was a significant predictive factor for grade 3–4 toxicity. This is in contradiction with our results, which show that low SMM is not a predictive factor for cetuximab DLT. However, the patients in the study by Barret et al*.* received cetuximab in combination with another chemotherapeutic agent, most commonly oxaliplatin. This makes it difficult to determine whether the predictive value of low SMM applies to cetuximab treatment or the chemotherapeutic treatment it was combined with. Both studies differ substantially in patient, tumor and treatment characteristics. In our study patients received concomitant radiotherapy which may also affect toxicity. Patients in the colorectal cancer study had metastatic disease and patients in our head and neck cancer study were unfit for cisplatin chemotherapy. These differences could be responsible for the fact that Barret et al., contrary to our study, concluded that low SMM was a predictive factor for DLT [10]. There are several hypotheses explaining the influence of low SMM on the occurrence of chemotherapy toxicity. Some hypothesize that the altered fat-to-lean body composition may influence the pharmacokinetics of chemotherapeuticals [19]. In HNSCC patients, low SMM appears to be independently associated with frailty [20], which describes a general state of increased vulnerability to stressors, such as cancer and anticancer treatment, and a higher risk of adverse events [13, 19, 20]. However, the hypothesis most supported in literature is based on the influence of low SMM on the drug distribution. The body is comprised of two major compartments, fat mass (FM) and lean body mass (LBM). Distribution of hydrophilic drugs, e.g. cisplatin, occurs mostly in the LBM, of which muscle mass is a large contributor [9, 19]. Therefore, a decrease in LBM due to low SMM may result in increased plasma levels and thereby increased risk of toxicity [3, 9, 17, 19, 22].

Low SMM has been demonstrated to have different predictive value for a variety of chemotherapeutical agents. This difference in predictive value could be explained by the mechanism of action by which low SMM causes an increased risk for toxicity. Platinum-based chemotherapies, such as cisplatin, carboplatin, and oxaliplatin, mostly distribute to the LBM and are, therefore, affected by the decrease in LBM in patients with low SMM [3]. Although cetuximab is also hydrophilic it has a very high molecular weight and, therefore, cetuximab distributes less towards the LBM and is mostly present in the plasma levels [23]. In the case of a patient with low SMM, it is possible that the decrease in LBM will not affect the plasma levels of cetuximab and, therefore, not increase the risk of toxicity. To be able to confirm this hypothesis additional research is needed.

Currently, it is unknown what the underlying pathophysiology of decreased SMM is, although there is a range of theories. First, it is hypothesized that age plays an important role in the mechanism of sarcopenia and decreasing SMM. This could be explained by the decrease of physical activity, the decrease of food intake, or the hormonal changes which are associated with aging [24]. Second, intracellular oxidative stress is speculated to be of influence on the occurrence of sarcopenia, specifically the increased concentration of inflammatory cytokines [12, 24]. Finally, there are theories about genetic components that could cause a decrease in SMM or muscle function [24]. Additional research into the mechanisms causing loss of muscle mass could progress the strategies for improving muscle mass and function, thereby improving overall survival. Further knowledge regarding drug distribution of chemotherapeutic agents could provide a better understanding of the process by which low SMM could cause an increased risk of toxicity. If there is a link between the distribution of a drug and the predictive value of low SMM for DLT, it would be possible to select a chemotherapeutic agent with less distribution towards the LBM or adapt the dose for patients with low SMM. This could result in less toxicity for patients with low SMM, however, it should not reduce the efficacy of the treatment. To ensure that efficacy is not reduced further research is required. To accurately determine whether patients with low SMM would profit more from treatment with cetuximab as opposed to cisplatin, a randomized controlled trial with endpoints toxicity and survival would be required.

## Conclusion

In conclusion, in contrast with cisplatin dose-limiting toxicity, low SMM has no predictive value for cetuximab dose-limiting toxicity in HNSCC patients treated with cetuximab and radiotherapy, probably attributable to the difference in lean body mass distribution of these chemotherapeutical agents. This study showed no significant prognostic value of low SMM for overall survival in HNSCC patients treated with cetuximab and radiotherapy unfit for platin-based chemotherapy.

## Abbreviations

SMM: Skeletal muscle mass
DLT: Dose-limiting toxicity
OS: Overall survival
DFS: Disease-free survival
LSMI: Lumbar skeletal muscle index
HU: Hounsfield units
C3: Cervical vertebra 3
L3: Lumbar vertebra 3
CSA: Cross sectional area
LBM: Lean body mass
HNSCC: Head and neck squamous cell carcinoma
